# Gene Expression Comparison between Alcohol-Exposed versus Not Exposed Pancreatic Ductal Adenocarcinoma Patients Reveals a Peculiar TGFβ-Related Phenotype: An Exploratory Analysis

**DOI:** 10.3390/medicina59050872

**Published:** 2023-04-30

**Authors:** Antonio Doronzo, Letizia Porcelli, Donatello Marziliano, Gianfranco Inglese, Antonella Argentiero, Amalia Azzariti, Antonio Giovanni Solimando

**Affiliations:** 1 U.O.C. Oncologia—Ospedale Mons. R. Dimiccoli, 76121 Barletta, Italy; 2 Laboratory of Experimental Pharmacology, IRCCS Istituto Tumori “Giovanni Paolo II” of Bari, 70124 Bari, Italya.azzariti@oncologico.bari.it (A.A.); 3 Guido Baccelli Unit of Internal Medicine, Department of Precision and Regenerative Medicine and Ionian Area—(DiMePRe-J), School of Medicine, Aldo Moro University of Bari, 70124 Bari, Italy; 4 Medical Oncology Unit, IRCCS Istituto Tumori “Giovanni Paolo II” of Bari, 70124 Bari, Italy; a.argentiero@oncologico.bari.it

**Keywords:** pancreatic cancer, PDAC, history of alcohol, TGFβ, fibrotic stroma

## Abstract

*Background*: Over the past few decades, there has been much debate and research into the link between alcohol consumption and the development and progression of pancreatic ductal adenocarcinoma (PDAC). *Objectives*: To contribute to the ongoing discussion and gain further insights into this topic, our study analysed the gene expression differences in PDAC patients based on their alcohol consumption history. *Methods*: To this end, we interrogated a large publicly available dataset. We next validated our findings in vitro. *Results*: Our findings revealed that patients with a history of alcohol consumption showed significant enrichment in the TGFβ-pathway: a signaling pathway implicated in cancer development and tumor progression. Specifically, our bioinformatic dissection of gene expression differences in 171 patients with PDAC showed that those who had consumed alcohol had higher levels of TGFβ-related genes. Moreover, we validated the role of the TGFβ pathway as one of the molecular drivers in producing massive stroma, a hallmark feature of PDAC, in patients with a history of alcohol consumption. This suggests that inhibition of the TGFβ pathway could serve as a novel therapeutic target for PDAC patients with a history of alcohol consumption and lead to increased sensitivity to chemotherapy. Our study provides valuable insights into the molecular mechanisms underlying the link between alcohol consumption and PDAC progression. *Conclusions*: Our findings highlight the potential significance of the TGFβ pathway as a therapeutic target. The development of TGFβ-inhibitors may pave the way for developing more effective treatment strategies for PDAC patients with a history of alcohol consumption.

## 1. Introduction

Integrating multi-omics data with clinical data at different molecular levels and epidemiological risk stratification represents an accurate and promising methodology able to resolve the complexity intrinsic to the biological systems characterising human pathology, including cancer. Specifically, pancreatic adenocarcinoma (PDAC) is characterised by genetic heterogeneity and variable aggressive behavior [1,2]. Pancreatic ductal adenocarcinoma is nowadays the seventh cause of cancer-related death. Even though several other malignancies still carry significant morbidity and mortality, prognosis has improved thanks to advances in treatment. Unfortunately, PDAC is an exception, with 5-year survival rates estimated between 10% and 30% in advanced and resected diseases, respectively [3]. Significant risk factors for developing pancreatic ductal adenocarcinoma encompass family history, obesity genetic disorders, diabetes mellitus (DM), chronic pancreatitis, intraductal papillary mucinous neoplasms and alcohol exposure [4]. Among the risk factors, alcohol consumption increased the risk of developing PDAC 1.22-fold in heavy drinkers (>37.5 g/day). Conversely, on-heavy or occasional drinkers (less than 37.5 g/day) showed no increase in the risk of pancreatic cancer [5]. Diagnosis is attained by fine needle aspiration sampling. Among described morphological variants, the most common histological entity is tubular adenocarcinoma. Poor prognosis owes to the inability to develop a strategy that allows the early identification in patients to detect the disease when intervention can improve survival.

With the extensive use of new technological platforms, it is possible to obtain many multi-parametric data by analysing the available databases [6,7,8,9].

The study aimed to dissect, at a gene expression level, the different phenotypes of PDAC arising from patients with a documented history of alcohol to identify distinctive transcriptional clusters with clinical implications. The analysis showed significant differences in gene expression between the two populations involving specific cellular pathways. In particular, through functional enrichment analysis, the genetic expression profile showed enrichment in the TGFβ-pathway. According to recent studies, this expression profile might represent one of the molecular drivers in the excessive production of fibrotic stroma through fibroblast activation in the tumor microenvironment [10]. This element seems closely related to neoplastic growth and the acquisition of resistance to chemotherapeutic treatments. These results would also support the clinical use of specific drugs in combination with traditional chemotherapy, as demonstrated in several clinical studies.

## 2. Experimental Section

### 2.1. Materials and Methods

#### 2.1.1. Determining Patient Cohorts

Statistical analysis was defined as a “not-low-moderate alcoholic history-onset” and “heavy alcoholic history-onset” (>30 g of alcohol per day) group of patients at the time of diagnosis. According to a recent study, it was hypothesized that low and moderate alcoholic exposure is not associated with pancreatic cancer risk [11,12], so they will be considered as not exposed.

Categorical variables were reported as percentages and compared using the Chi-squared or Fisher’s exact test when needed. Time-to-event outcomes (mortality) were evaluated using the Kaplan–Meier method. Statistical significance was set at *p* < 0.05. All statistical analyses were performed using SPSS software (IBM SPSS software, Chicago, IL, USA; Version 24.0).

#### 2.1.2. TCGA Cohort

To reach the purpose of the study, the TGCA (The Cancer Genome Atlas) dataset [13]. Eligible patients were those who were defined as having PDAC in the TCGA dataset and who had complete information on sex, age at PDAC diagnosis, tumor histology, alcohol history, DNA analysis and gene expression profiles.

#### 2.1.3. Cell Culture

MiaPaCa-2 cell line from an undifferentiated human pancreatic carcinoma was purchased from ATCC. MiaPaCa-2 cells were grown as recommended by the supplier.

#### 2.1.4. Detection of TGFβ Receptor by Flow Cytometry (FCM)

MiaPaCa-2 cells were seeded at a density of 3 × 10^5^/well in 6-well plates and incubated at 37 °C and 5% CO_2_ to allow attachment. Then, 7nM Et-OH was added daily in each well, and the plates were incubated for 8 days. Afterwards, the cells were harvested, washed twice, resuspended in ice-cold PBS without Ca^2+^ and Mg^2+^, fixed in Ethanol 70% and stored at −20 °C O.N. After centrifugation, cells were stained as reported in [14]. Cells were analyzed using an Attune NxT Acoustic Focusing Cytometer (Thermo Fisher Scientific, Waltham, MA, USA) and Attune™ NxT Software 3.1.1162.1 (Thermo Fisher Scientific, Waltham, MA, USA).

## 3. Results

### 3.1. Patients

From the TGCA cohorts, *n* = 171 patients have been included in the analysis: *n* = 66 not alcohol-exposed (NAE), and *n* = 105 heavy alcohol-exposed patients (HAE). There was a slight male predominance in the cohort (95/171; 55%), though not statistically significant. A total of 105 patients were <70 years old, 66 were ≥70 years old and in HAE group, 66 of patients were <70 years old (66/171; 38%). The most common stage was IIb (115/171; 67%), while the most common histotype was ductal adenocarcinoma (139/171; 81%). The primary tumor location was the head of the pancreas for 81% of both groups. There were increased rates of pancreatitis in HAE patients (72/105; 69%) but not DM in each group. Moreover, the rate of DM approached 50% in this cohort. This value seems to be highly dependent on the surveyed population. It significantly varies among studies, encompassing rates from 20% in an european cohort (*p* = 0.778) [15], reaching 35–40% (*p* = 0.76) [16], up to 68% [17]. This research has been approved by IRCCS Cancer Institute “Giovanni Paolo II” of Bari ethic committee, approved on 31 December 2019, with Prot n. 806/EC and was activated with resolution no. 1011/2019.

The patient characteristics of the cohort are presented in Table 1.

### 3.2. Genomic Landscape

RNAseq data are available from the TCGA cohort pancreatic tumors to further characterize the differences between tumor gene expression signatures and genes. A statistically significant gene list was obtained comparing the gene-expression profiling from alcohol-exposed versus not exposed PDAC patients (*p* < 0.05, FDR < 5). The complete gene list (*n* = 142) included 113 tumor suppressor genes and 29 oncogenes.

The genes list is presented in Table 2 and Table 3.

**Figure 1:**
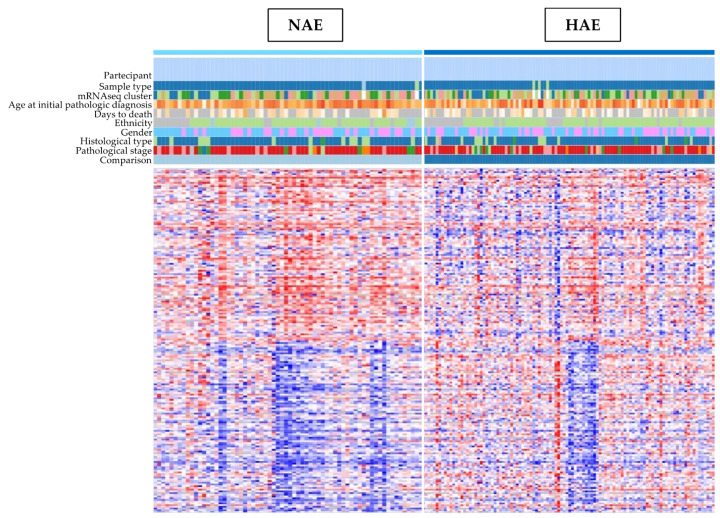
Heat Maps of differentially expressed genes in two populations NAE and HAE with different characteristics regarding sex, age, gender, year of diagnosis and histopathological. Red, upregulated genes; blue, downregulated genes. Morpheus is a flexible matrix visualization and analysis software that allows for uncovering a given dataset in a heat map. With its interactive tools, it is possible to deep-dive into data by clustering, generating new annotations, searching, filtering, sorting, and displaying charts, among other features. This versatile software enables a comprehensive understanding and analysis of your data, making it an invaluable resource for various applications. Details are available at Morpheus: https://software.broadinstitute.org/morpheus, accessed on 28 December 2022.

The following is a heat map of differentially expressed genes correlated with the alcohol exposure of PDAC patients. There were 171 samples in both the NAE and HAE survival groups. Based on t-test analysis in Morpheus with a *p* ≤ 0.01 criterion, a total of 1000 genes (500 upregulated genes and 500 downregulated genes) were identified (Figure 1).

The entire list of the selected genes was used to build up a biological network for functional network enrichment, which enhanced the biological process using Kyoto Encyclopedia of Genes and Genomes—Genome (KEGG) and STRING database (The STRING database in 2017: quality-controlled protein-protein association networks, made broadly accessible).

**Figure 2:**
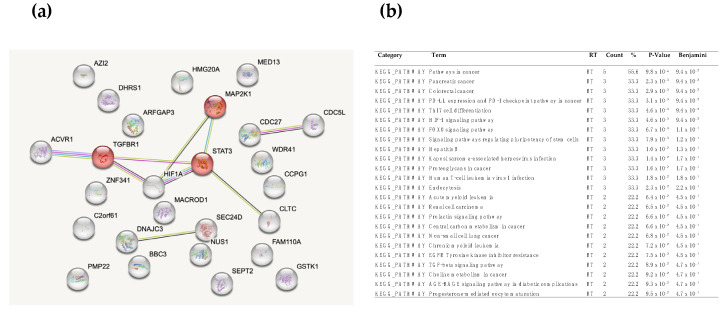
TGFβR1 signaling pathway upregulated in patients’ gene signature with alcoholic history, built up with STRING (**a**) and KEGG databases (**b**).

Among the tested signatures, the TGFβ signaling pathway was identified as statistically associated with alcoholic history (Figure 2).

**Figure 3:**
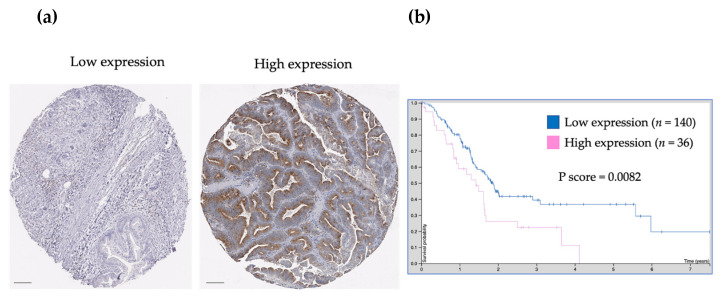
TGFβR1 representative expression by immunohistochemistry (**a**) and patient survival (**b**). In B, the Log-rank *p* value for the Kaplan–Meier plot shows results from the analysis of the correlation between mRNA expression level and patient survival in the Human Protein Atlas. Scale bar 100 mcm.

These results fit and validate the relationship between TGFβR1 expression (low and high) and patient survival in pancreatic cancer (Figure 3, from Human Protein Atlas).

### 3.3. In Vitro Proof of Concept Validation

**Figure 4:**
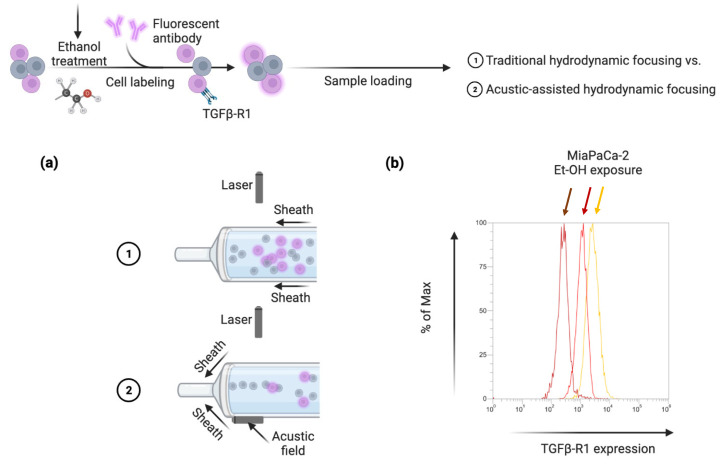
Scheme of TGFβR1 detection by FCM (**a**) and results of Et-OH-treated MiaPaCa-2 cell line (**b**). Brown arrow indicates unstained cells, red arrow untreated cells and yellow arrow Et-OH treated cells.

To confirm the obtained results in an in vitro PDAC model, we investigated whether there was an upregulation of TGFβR1 in MIA-PaCa cells after treatment with Et-OH compared to controls (Figure 4).

Cells are treated with Et-OH at 7 nM, the maximum alcohol consumption allowed in the U.S. for 8 days [18]. After Et-OH exposure, cells showed a strong increase of TGFβR1 compared to untreated cells.

## 4. Discussion

Pancreatic cancer remains one of the major causes of cancer-related death worldwide. Complete removal by surgery is the primary therapeutic option in the early stage for the best cure rate. However, the mortality rate remains high for patients diagnosed with pancreatic cancer at advanced stages despite surgery, radiotherapy and chemotherapy, with survival rates approaching 10% and 30% for advanced and resected disease, respectively at 5 years [19]. Novel approaches, including targeted therapies based on molecular profiling of pancreatic cancer, as well as the improvement of surgical techniques with a reduction in surgical morbidity, have improved survival in several cases of resectable and advanced disease. Therefore pancreatic cancer management is moving towards a multidisciplinary approach [2]. 

Interestingly, our bioinformatic analysis showed significant differences in gene expression between the two populations (not alcohol-exposed and heavy alcohol-exposed) involving specific cellular pathways. In particular, through functional enrichment analysis, the genetic expression profile showed enrichment in the TGFβ-pathway.

In the frame of thinking expressed by recent studies, this expression profile might be one of the pivotal molecular drivers of the excessive production of fibrotic stroma through fibroblast activation in the tumor microenvironment.

Indeed, pancreatic cancer develops in a microenvironment, and the stroma, enriched with extracellular matrix proteins, are mainly produced by pancreatic stellate cells (PSCs) known as cancer-associated fibroblasts (CAFs), inflammatory cells such as mast cells (MC), and small blood vessels, which recent evidence suggests are a dynamic compartment rather than a mechanical barrier intensely involved in the process of tumor formation, progression, invasion and metastasis [20,21]. The paracrine crosstalk of tumor and stroma cells has been demonstrated to play a pivotal role in tumor cells’ transformation, and recently, even in chemoresistance [22]. In vitro, evidence suggested that among stroma cells, CAFs played a significant role in the acquisition of the hallmarks of pancreatic cancer, including chemoresistance [23,24], whereas the presence of inflammatory cells, such as mast cells infiltrating pancreatic cancer, has been associated with a worse prognosis because it promoted angiogenesis, which is the development of the desmoplastic microenvironment and tumor invasion [25].

Moreover, it has been demonstrated that PSCs differentiate into myofibroblasts in pancreatic fibrosis PDAC [26]. PSCs have been shown to play a crucial role in chronic pancreatitis leading to fibrosis. During chronic pancreatitis, strongly associated with alcoholic exposure, PSCs are activated [27] by acinar and immune cells in a paracrine way through the secretion of TGF-β [10,28]. Additionally, cytokines, reactive oxygen species (ROS), and oxidative stress in the fibrotic areas of pancreatitis contribute to PSC activation [27]. Furthermore, chronic pancreatitis gives a high risk for PDAC development, indicating the role of the fibrotic microenvironment in PDAC progression.

An aPSC-induced desmoplastic reaction plays a significant role in chemoresistance. The extensive desmoplastic reaction with an abundant amount of aPSC-secreted ECM proteins leads to intratumoral hypoxia and a self-perpetuating fibrosis cycle [29]. Tumoral hypoxia causes genomic instability of cancer cells leading to epithelial-to-mesenchymal transition (EMT), increased malignant behaviour, and resistance to chemotherapy [29,30].

It was demonstrated that the crosstalk between MS, CAFs, and PDAC cells strongly reduced the Gemcitabine–NabPaclitaxel dependent inhibition of tumor cell viability through the activation of TGFβ-signaling, and that the selective inhibition of TGβR1 receptor by galunisertib, a specific inhibitor, restored the sensitivity to chemotherapy drugs and could be used in combination with gemcitabine to improve patient outcomes, as demonstrated in several studies [31,32].

These pieces of evidence demonstrate that aPSCs and CAFs exacerbate the EMT, not only by producing ECM, but also by establishing crosstalk with cancer cells and other stromal cells. Thus, disrupting the crosstalk using targeting technologies or modulating the tumor stroma may provide novel therapeutic options.

This study has clear limitations, mainly due to the need of confirmation in statistically powered prospective observation. Moreover, we observed a certain degree of variability in reported rates of DM prevalence, even when looking at similar populations [16,17]. Indeed, DM is a common comorbidity in pancreatic ductal adenocarcinoma (PDAC) patients. The TGCA (The Cancer Genome Atlas) cohort is a large dataset of PDAC patients that has been extensively studied for various aspects of the disease. One interesting finding from the TGCA cohort is that the rate of DM is greater than 50%, which is higher than the rates reported in other cohorts. For example, two studies conducted in Asian populations reported a risk of DM at around 35–40% in PDAC patients [16,33]. On the other hand, a study conducted in a European cohort reported a rate of DM at around 20% [15]. The difference in the rate of DM in various cohorts could be due to several factors. For instance, differences in the ethnicity, lifestyle, and genetic makeup of the cohorts could contribute to the variation in the rates of DM. Additionally, the methods used to diagnose DM, and the criteria used to define it, could also influence the reported rates.

The high rate of DM in the TGCA cohort is noteworthy, as it indicates that PDAC patients in this cohort may have a higher prevalence of glucose intolerance and insulin resistance. These factors could have implications for the management of PDAC patients, as glucose intolerance and insulin resistance could affect treatment outcomes and increase the risk of complications. Therefore, acknowledging and discussing the difference in the rate of DM in various cohorts, is important for a comprehensive understanding of PDAC and its associated comorbidities. It highlights the need for further research to investigate the underlying mechanisms that contribute to the differences in DM rates, and to develop effective management strategies for PDAC patients with comorbid DM. It’s important to consider the specific characteristics of the population being studied, as well as the methods used to measure DM, in order to accurately interpret and compare results across studies [34]. Additionally, the high prevalence of DM in this cohort underscores the importance of continued research and interventions to prevent and manage this chronic disease. Nonetheless, it paves the way for a growing attention to patients with a documented history of alcohol exposure, and therefore, our study corroborated the hypothesis that solid stiffness in PDAC and subsequently decreasing solid stress, holds the potential for therapeutic targeting. ECM components, such as collagen, hyaluronic acid, and aPSCs, are the main components of the stroma causing substantial stress [12,27]. A few studies have investigated the effect of the stroma and/or stromal components on drug penetration. Other studies have enzymatically degraded hyaluronic acid in the tumor stroma, which resulted in normalized interstitial fluid pressure, re-expansion of the vasculature, increased tumor suppression with gemcitabine, and prolonged survival [35]. PEGylated hyaluronidase (PEGPH20) has been assessed with gemcitabine, improving survival and attenuating tumor growth in mice compared with gemcitabine alone, by improving progression-free and overall survival rates of patients with metastatic PDAC [36]. PEGPH20 is currently in clinical trials in patients with advanced cancer to better tailor personalized treatment based on novel biomarkers [6]. TGFβ (transforming growth factor beta) is a crucial regulator of cell growth and differentiation, and it has been studied extensively in the context of various diseases, including cancer. MIA-PaCa-2 cells, which are derived from the human pancreas, are known to express TGFβ. The expression of TGFβ in MIA-PaCa-2 cells has been studied in the context of pancreatic cancer. Studies have shown that MIA-PaCa-2 cells express higher levels of TGFβ than normal pancreatic cells. This suggests that TGFβ may contribute to the progression of pancreatic cancer. However, it is unclear exactly how TGFβ contributes to the progression of pancreatic cancer in MIA-PaCa-2 cells. Studies have also shown that specific signaling pathways modulate TGFβ expression in MIA-PaCa-2 cells. For example, the Wnt signaling pathway has been shown to upregulate TGFβ expression in MIA-PaCa-2 cells. This suggests that the Wnt pathway may be involved in the progression of pancreatic cancer in MIA-PaCa-2 cells with implications for immune targeting [37]. In addition, TGFβ expression in MIA-PaCa-2 cells is regulated by other factors, such as microRNAs [38]. Our data suggest that alcohol may contribute to the progression of pancreatic cancer by upregulating the expression of TGFβ [39]. However, it is not clear exactly how alcohol increases the expression of TGFβ in these cells. Further research is needed to determine the exact mechanisms by which alcohol may increase the expression of TGFβ in MIA-PaCa-2 and PANC1 cells, paving the way for novel therapies [40,41,42,43,44].

## 5. Conclusions

Pancreatic cancer remains a challenging disease to treat with high mortality rates despite advancements in surgery, radiotherapy, and chemotherapy. Resistance to chemotherapy heavily affects the clinical outcome of patients. Herein, it first uncovered the overexpression of TGFβ-pathway in patients with a documented history of alcohol consumption. Targeted approaches based on molecular profiling, such as the inhibition of TGFβ signaling and improvement of surgical techniques, may improve patient outcomes. Moreover, the high prevalence of DM in pancreatic cancer patients highlights the need for continued research and interventions to prevent and manage this chronic disease. Future studies with larger sample sizes and statistically powered prospective observations are needed to confirm the findings of this study and pave the way for personalized treatment options based on novel biomarkers. The results validate the potential role of TGF-β pathway and tumor stroma as therapeutic targets for PDAC providing a personalized therapeutic strategy. 

## Figures and Tables

**Table 1:** Patient characteristics from TCGA dataset.

TCGA					
		Not Alcohol Exposed (*n* = 66)	%	Heavy Alcohol Exposed (*n* = 105)	%
Gender	Female	27	41	48	46
	Male	39	59	57	54
Age	<70	39	59	66	63
	≥70	27	41	39	37
Stage	I	9	13	13	12
	II	54	81	86	83
	III	0	0	3	3
	IV	4	6	2	2
Istotype	Ductal adenocarcinoma	52	79	87	83
	Adenocarcinoma other types	13	20	13	12
	Colloid-mucinous carcinoma	1	1	3	3
	Indifferenziate carcinoma	0	0	2	2
Location	Head	55	83	90	86
	Body	10	15	10	9
	Tail	1	2	5	5

**Table 2:** Oncogene List from https://www.proteinatlas.org/ *.

Gene	Med Exp	Med FU	P Score	5 y OS Hi	5 y OS Low	Prognostic Cancer
**C2ORF61**	0.18	1.27	0.0024	16	35	
**BBC3**	4.57	1.27	0.072	23	31	Urothelial, Endometrial
**HIF1A**	38.46	1.27	0.018	0	35	None
**AGFG1**	8.37	1.27	0.02	0	37	Liver, Lung
**DNAJC3**	19.2	1.27	0.12	25	41	Endometrial
**CDC27**	7.64	1.27	0.004	7	38	Renal, Liver
**DHRS1**	6.38	1.27	0.0022	20	31	Liver, Lung
**CDC5L**	7.18	1.27	0.0063	16	37	Melanoma
**HMG20A**	3.28	1.27	0.046	15	44	None
**AZI2**	2.82	1.27	0.27	13	38	Liver, Urothelial
**MAP2K1**	15.15	1.27	0.14	20	47	Glioma
**WDR41**	3.85	1.27	0.0023	0	35	Liver
**FAM110A**	5.7	1.27	0.023	19	31	Renal
**GSTK1**	35.23	1.27	0.02	25	33	Renal, Breast
**ARFGAP3**	22.65	1.27	0.0091	15	44	None
**NUS1**	5.73	1.27	0.26	13	36	Cervical
**GTPBP8**	2.11	1.27	0.014	18	46	Renal
**PMP22**	36.19	1.27	0.077	14	48	Renal
**ZNF341**	1.39	1.27	0.074	19	30	Renal
**STAT3**	27.99	1.27	0.22	16	40	Pancreatic
**SEC24D**	8.06	1.27	0.12	9	37	Renal
**CCPG1**	3.39	1.27	0.032	11	40	Renal
**ACVR1**	14.78	1.27	0.026	0	41	Urothelial
**MACROD1**	6.89	1.27	0.00029	20	33	Pancreatic
**SEPT2**	56.41	1.27	0.0011	0	42	Liver
**MED13**	5.56	1.27	0.19	0	36	Colon
**CLTC**	31.53	1.27	0.033	18	33	Urothelial, Liver
**TGFβ-R1**	12.42	1.27	0.0082	0	37	Pancreatic

* Accessed on 28 December 2022.

**Table 3:** Oncosuppressor gene list; from https://www.proteinatlas.org/ *.

Gene	Med Exp	Med FU	P Score	5 y OS Hi	5 y OS Low	Prognostic Cancer
**PAOX**	2.51	1.27	0.59	35	24	Head, Renal, Cervical
**TYSND1**	5.14	1.27	0.56	39	14	None
**ZNF282**	9.87	1.27	0.033	40	6	Liver
**PSMG4**	1.23	1.27	0.57	37	18	Renal
**RGS14**	6.59	1.27	0.27	36	14	Liver, Glioma
**PWWP2B**	12.03	1.27	0.13	36	0	Renal
**TMUB1**	22.77	1.27	0.017	41	0	None
**ACTR5**	4.02	1.27	0.05	36	0	Liver, Renal
**FDXR**	3.17	1.27	0.11	42	8	Endometrial
**UBAC1**	12.07	1.27	0.0039	35	10	Renal, Cervical
**AGAP3**	8.3	1.27	0.029	40	8	Liver, Colon
**EEFSEC**	8.26	1.27	0.016	38	8	Cervical
**SLC25A22**	7.03	1.27	0.025	37	0	None
**NTHL1**	6.72	1.27	0.028	45	8	None
**MGC70857**	6.82	1.27	0.36	38	16	Renal
**C7orf47**	15.2	1.27	0.068	44	17	Urothelial
**AMDHD2**	3.63	1.27	0.019	39	18	Cervical
**PDK4**	15.85	1.27	0.019	46	25	Stomach
**CTU1**	1.61	1.27	0.04	42	8	Urothelian
**TRAF2**	8.64	1.27	0.074	38	10	Renal, Colon
**CHCHD1**	18.75	1.27	0.45	32	19	Urothelial
**ASAH2B**	0.79	1.27	0.038	34	0	None
**GHR**	0.62	1.27	0.0061	37	24	Liver
**GLIPR1L1**	0.17	1.27	0.0079	54	16	None
**CCDC61**	4.56	1.27	0.0027	40	0	None
**COBRA1**	31.99	1.27	0.005	37	6	Liver
**EPB41L3**	2.54	1.27	0.0011	45	23	None
**YPEL3**	17.46	1.27	0.002	41	0	Head
**CCDC85B**	14.11	1.27	0.099	36	0	Renal
**IL6R**	3.62	1.27	0.00017	38	21	Pancreatic
**FAM46A**	5.99	1.27	0.0014	35	0	Renal
**RNF208**	5.15	1.27	0.0084	38	0	Renal
**CYHR1**	6.17	1.27	0.0071	44	9	Live, Colon
**NUDT6**	0.32	1.27	0.063	39	11	None
**SCRIB**	14.39	1.27	0.082	39	0	None
**ANKRD13D**	6.13	1.27	0.0051	41	0	Renal
**HAUS5**	2.78	1.27	0.016	41	6	Renal, Liver
**DBP**	1.89	1.27	0.0042	41	0	Renal, Lung
**LAS1L**	6.76	1.27	0.21	46	20	None
**TMEM160**	8.02	1.27	0.0082	40	0	None
**SNAPC2**	12.64	1.27	0.055	38	8	None
**ZNF517**	1.95	1.27	0.00017	42	0	Pancreatic
**HAGHL**	1.67	1.27	0.064	37	0	Renal
**NUDT22**	7.25	1.27	0.076	38	0	Renal
**ZNF219**	4.18	1.27	0.06	39	0	None
**PRMT7**	3.11	1.27	0.0047	41	6	Endometrial
**LRRC45**	5.15	1.27	0.0062	40	0	Renal
**TMCO6**	3.31	1.27	0.028	41	7	Renal
**NDUFV1**	28.07	1.27	0.04	38	0	Renal
**C8orf44**	1.43	1.27	0.0042	43	0	Renal, Pancreatic
**ZNF511**	4.44	1.27	0.014	37	0	Renal
**TIGD5**	2.39	1.27	0.0028	39	0	Renal, Liver
**PSMG3**	15.83	1.27	0.27	35	0	Liver
**GLI4**	4.19	1.27	0.0075	43	7	None
**RPUSD1**	7.64	1.27	0.2	39	8	None
**METT11D1**	7.77	1.27	0.14	41	13	None
**SLC9A8**	5.13	1.27	0.016	37	17	None
**GFER**	7.73	1.27	0.16	43	8	Renal
**SNRNP70**	41.38	1.27	0.000046	47	0	Pancreatic, Renal
**ABTB1**	8.59	1.27	0.0046	37	6	Renal
**FAM173A**	6.04	1.27	0.04	38	0	Renal
**SIGIRR**	14.29	1.27	0.023	37	13	Renal, Urothelial
**FAM120B**	5.44	1.27	0.0027	32	0	Pancreatic
**SPSB3**	0.75	1.27	0.034	51	19	None
**LRRC20**	4.25	1.27	0.094	39	7	Renal
**CLU**	103.03	1.27	0.029	45	20	Tyroid
**PDDC1**	11.56	1.27	0.0052	38	0	Renal, Liver
**FASTK**	20.17	1.27	0.0004	42	0	Pancreatic, Colon
**PARP10**	13.3	1.27	0.17	43	25	None
**ADCK5**	4.64	1.27	0.066	39	0	None
**AQP7**	0.61	1.27	0.00086	48	12	Renal
**PLDN**	7.24	1.27	0.047	32	0	None
**D2HGDH**	5.56	1.27	0.066	39	16	Renal
**FNDC3A**	10.7	1.27	0.019	34	0	Renal
**MRPS26**	26.56	1.27	0.0096	50	8	None
**FBXW5**	36.29	1.27	0.0035	40	7	Renal, Endometrial
**COMTD1**	11.76	1.27	0.094	36	8	Renal
**MRPS25**	5.87	1.27	0.063	43	23	Renal
**CWF19L1**	5.02	1.27	0.027	39	0	Liver
**NPEPL1**	2.27	1.27	0.0016	40	0	Renal
**RAPGEF4**	0.92	1.27	0.00013	36	22	Pancreatic
**CDK5**	5.27	1.27	0.0062	34	0	None
**ACAD10**	2.91	1.27	0.0062	51	14	Renal
**MTG1**	1.66	1.27	0.00051	44	0	Pancreatic, Renal
**CENPB**	39.3	1.27	0.0015	47	7	Liver
**DNAJB9**	12.66	1.27	0.18	37	24	None
**NDOR1**	4.7	1.27	0.0026	47	16	None
**SLC27A1**	5.69	1.27	0.11	36	16	Renal
**ZNF212**	5.02	1.27	0.0043	44	6	None
**TPPP3**	13.3	1.27	0.001	34	14	Renal
**H1FX**	64.28	1.27	0.0053	39	0	None
**ANAPC4**	3.34	1.27	0.13	34	20	Renal, Urothelial
**INTS1**	13.38	1.27	0.00048	42	5	Pancreatic, Liver
**KLHDC4**	2.91	1.27	0.00011	51	6	Pancreatic
**CHCHD10**	19.27	1.27	0.16	33	15	Renal
**FAM98C**	4.53	1.27	0.028	43	7	Ovarian, Urothelial
**XYLT2**	7.87	1.27	0.015	49	19	None
**NME3**	28.19	1.27	0.044	45	14	Breast
**BCL7A**	3.06	1.27	0.31	44	21	Renal, Liver
**TSNARE1**	3.77	1.27	0.0069	38	0	Urothelial
**FBXL8**	2.69	1.27	0.011	39	0	Endometrial
**EIF1AY**	1.03	1.27	0.042	42	23	None
**C4orf23**	1.39	1.27	0.0034	35	15	None
**PRKRIP1**	8.21	1.27	0.0026	50	13	Renal, Urothelial
**C8orf42**	2.18	1.27	0.00094	51	11	Pancreatic, Endometrial
**ZNF579**	4.88	1.27	0.0058	40	0	Renal
**C5orf45**	2.15	1.27	0.0036	56	16	Renal
**PSMD9**	2.19	1.27	0.15	48	16	Liver
**SELO**	10.81	1.27	0.0082	39	0	Urothelial
**BAD**	21.8	1.27	0.14	37	0	None
**C9orf69**	14.24	1.27	0.063	40	11	Endometrial

* Accessed on 28 December 2022.

## Data Availability

The data are unavailable due to privacy.
